# Genetic Burden for Late-Life Neurodegenerative Disease and Its Association With Early-Life Lipids, Brain, Behavior, and Cognition

**DOI:** 10.3389/fpsyt.2020.00033

**Published:** 2020-02-07

**Authors:** Sander Lamballais, Ryan L. Muetzel, Mohammad Arfan Ikram, Henning Tiemeier, Meike W. Vernooij, Tonya White, Hieab H. H. Adams

**Affiliations:** ^1^Department of Epidemiology, Erasmus MC University Medical Center Rotterdam, Rotterdam, Netherlands; ^2^Department of Child and Adolescent Psychiatry and Psychology, Erasmus MC University Medical Center Rotterdam, Rotterdam, Netherlands; ^3^Department of Social and Behavioral Science, Harvard T. H. Chan School of Public Health, Boston, MA, United States; ^4^Department of Radiology and Nuclear Medicine, Erasmus MC University Medical Center Rotterdam, Rotterdam, Netherlands; ^5^Department of Clinical Genetics, Erasmus MC University Medical Center Rotterdam, Rotterdam, Netherlands

**Keywords:** polygenic risk scores, Alzheimer’s disease, Parkinson’s disease, frontotemporal dementia, cognition, neuroimaging, lipid profiles, internalizing behavior

## Abstract

**Background:**

Genetics play a significant role in the etiology of late-life neurodegenerative diseases like Alzheimer’s disease, Parkinson’s disease, and frontotemporal dementia. Part of the individual differences in risk for these diseases can be traced back decades before the onset of disease symptoms. Previous studies have shown evidence for plausible links of apolipoprotein E (APOE), the most important genetic marker for Alzheimer’s disease, with early-life cognition and neuroimaging markers. We aimed to assess whether genome-wide genetic burden for the aforementioned neurodegenerative diseases plays a role in early-life processes.

**Methods:**

We studied children from the Generation R Study, a prospective birth cohort. APOE genotypes and polygenic genetic burdens for Alzheimer’s disease, Parkinson’s disease, and frontotemporal dementia were obtained through genome-wide genotyping. Non-verbal intelligence was assessed through cognitive tests at the research center around the age of 6 years, and educational attainment through a national school performance test around the age of 11 years. The Child Behavior Checklist was administered around the age of 10 years, and data from the anxious/depressed, withdrawn/depressed, and the internalizing behavior problems scales were used. Children participated in a neuroimaging study when they were 10 years old, in which structural brain metrics were obtained. Lipid serum profiles, which may be influenced by APOE genotype, were assessed from venal blood obtained around the age of 6 years. The sample size per analysis varied between 1,641 and 3,650 children due to completeness of data.

**Results:**

We did not find evidence that APOE genotype or the polygenic scores impact on childhood nonverbal intelligence, educational attainment, internalizing behavior, and global brain structural measures including total brain volume and whole brain fractional anisotropy (all p > 0.05). Carriership of the APOE ε2 allele was associated with lower and APOE ε4 with higher low-density lipoprotein cholesterol concentrations when compared to APOE ε3/ε3 carriers.

**Conclusion:**

We found no evidence that genetic burden for late-life neurodegenerative diseases associates with early-life cognition, internalizing behavior, or global brain structure.

## Introduction

Genetic factors play a significant role in the etiology of late-life neurodegenerative diseases like Alzheimer’s disease (AD) (1, 2), Parkinson’s disease (PD) (3), and frontotemporal dementia (FTD) (4). With the exception of rare Mendelian forms of diseases, cases arise due to multifactorial processes where many genetic variants confer risk of neurodegeneration, in combination with non-genetic factors. The clinical onset of the aforementioned diseases tends to be preceded by years of deterioration of cognition and brain structure (5–7) as well as an increased incidence of depressive and psychiatric symptoms (8–10). For AD, these differences may even extend decades before the onset of disease (11–13), which could partly be explained by individual differences in the genetic burden for AD. As the genome is stable throughout life, the genes implicated in late-life neurodegenerative disease may already lead to subtle differences during childhood.

The apolipoprotein E epsilon 4 allele (APOE ε4) is the strongest common genetic variant for AD (14–16). The APOE gene plays a role in lipoprotein metabolism, and has been shown to affect lipid serum profiles during adulthood (17–21), and potentially during childhood (22, 23). As APOE also increases the risk for AD, its role in early-life cognition and brain markers has also been studied. The studies on APOE ε4 and cognition during adolescence and early adulthood have reported mixed results, with some reporting lower cognitive function, some higher, and most reporting no difference (24). Additionally, a number of studies showed that APOE ε4 may relate to lower brain volumes during infancy and childhood, particularly in regions affected in AD such as the hippocampus (25–30). Overall, APOE ε4 may associate with early-life processes, but this needs to be elucidated further.

With the advent of genome-wide association studies (GWAS) there has been an increase in the number of genes identified for neurodegenerative disease. GWAS has led to the discovery of at least 30 new genetic loci for AD (1, 2), at least genetic 24 loci for PD (3), and at least 3 loci for FTD (4). The disease burden per locus can be combined into a single score, known as polygenic risk scores (PGRS), to assess the genetic burden a person has for that disease (31). The genetic burden for AD, PD, and FTD may relate to early-life processes, which can be studied using PGRS. However, few studies exist that assesses the effect of such PGRS on early-life markers.

To obtain a more comprehensive overview of the relevance in early-life of genes related to late-life neurodegenerative disease we performed a comprehensive study within the Generation R birth cohort. We assessed the APOE genotype and created PGRS for AD, PD, and FTD. Given the existing literature we hypothesized that these genetic predispositions to late-life neurodegenerative disorders associate with early-life non-verbal intelligence quotient (IQ), educational attainment, internalizing behavior, and neuroimaging markers, and that APOE and the AD PGRS associate with lipid profiles.

## Methods

### Participants

The data was obtained from the Generation R cohort, a prospective birth cohort based in Rotterdam, the Netherlands (32). Pregnant women in Rotterdam were at their first prenatal visit approached to participate. A total of 9,901 children were born as part of the Generation R cohort and were invited to participate in questionnaires and research center visits beginning in 2002 to the present day.

DNA was sequenced from blood obtained from the umbilical cord or with blood samples collected around 6 years of age, and genetic data was available for 5,725 children. In the case of sibling pairs (n = 235 pairs) we included the oldest sibling. This led to a sample of 5,490 children. The focus of the current study was on cognitive function, brain structure, and blood lipid profiles. Non-verbal IQ was measured at approximately 6 years (n = 3,650) and educational attainment at 11 years of age (n = 1,641). The Childhood Behavior Checklist (CBCL) was administered around the age of 10 years with data for the anxious/depressed scale (n = 1,867), the withdrawn/depressed scale (n = 1,862), and the internalizing problems scale (n = 1,859) used for this study. Magnetic resonance imaging (MRI) of the brain was done when the children were approximately 10 years of age, collecting both T_1_-weighted (n = 1,962) and diffusion-weighted images (n = 1,832). Blood lipid profiles were determined with blood samples obtained around the age of 6 years (n = 2,749). A flow chart of the study population is shown in **Supplementary Figure 1**.

### Ethics Statement

The study was conducted in accordance with the guidelines as proposed in the World Medical Association Declaration of Helsinki and was approved by the Medical Ethics Committee of the Erasmus MC. Written informed consent was obtained from primary caregivers on behalf of the child.

### Genotyping, Apolipoprotein E ɛ4, and Polygenic Risk Scores

DNA sample collection, genotype calling procedures, and subsequent quality control have been described elsewhere (33, 34). In brief, samples were either collected from cord blood at birth (Illumina 610K Quad Chip) or from venipuncture at a visit to the research center when children were between the age of 5 and 8 years (Illumina 660K Quad Chip). Single nucleotide polymorphisms were filtered for minor allele frequency < 0.01, Hardy-Weinberg disequilibrium p < .00001, and missing rate > 0.05. To be able to account for population stratification, we calculated the first 10 genomic components using the multi-dimensional scaling function of PLINK (34, 35).

APOE carriership status was assessed from the genotyped data and based on the nucleotide combinations of two single nucleotide polymorphisms: rs429348 and rs7412. A thymine at both locations is classified as APOE ε2, one thymine and one cytosine as APOE ε1 or APOE ε3, and both cytosines as APOE ε4. As APOE ε1 and APOE ε3 cannot be distinguished we classified both as APOE ε3. We considered APOE ε3/ε3 to be the reference category as this is the most prevalent genotype.

PGRS for AD, PD, and FTD were calculated using PRSice-2 (36). The scores were based on summary statistics from the largest GWAS for each respective neurodegenerative disease (3, 4, 37). PGRS are generally calculated for different thresholds of statistical significance in the summary statistics. As we did not have an *a priori* hypothesis on the optimal threshold, we calculated PGRS based on single nucleotide polymorphisms below the following p-value thresholds: 0.000001, 0.000005, 0.00001, 0.000005, 0.00001, 0.00005, 0.0001, 0.0005, 0.01, 0.05, 0.1, 0.5, and 1.0. Strand flips were corrected and we used clumping to build the score using independent loci.

### Non-Verbal Intelligence Quotient and Educational Attainment

Two measures for cognitive function were available. The first was an assessment of non-verbal IQ at the research visit around the age of 6 years. Participants completed two subtests of the Snijders-Oomen Non-Verbal Intelligence Test-Revised (SON-R 2½-7) (38): “Mosaics,” a spatial visualization task, and “Categories,” an abstract reasoning task. The raw scores were converted to IQ scores using age and sex-specific norms. As both tasks specifically assess non-verbal cognition, we considered these scores as non-verbal IQ scores. The correlation between IQ derived from the whole test battery and IQ derived from just the “Mosaics” and “Categories” tests has been shown to be high (r = 0.86) (39).

The measure of cognitive function was the educational attainment score obtained at the age of 11 years. The “Centraal Instituut voor Toetsontwikkeling” (CITO) test is administered in the majority primary schools in the Netherlands and is completed during the final year of primary school. The CITO test generally consists of two main skill domains: language and mathematics. The raw test scores for both domains were obtained for most Generation R children that took the CITO test during the years 2014 to 2017 and that were still part of Generation R at the time. As the test difficulty tends to vary slightly each year we summed the raw domain scores to a total score for each child, standardized the scores for all children within a given year, and finally combined the stratified distributions into one distribution. This method yielded standardized scores that were comparable across testing years.

### Child Behavior Checklist

Behavioral problems were assessed using the CBCL for ages 6 to 18 (40). The CBCL is a validated and reliable 113-item inventory that uses caregiver-reported information to assess behavioral problems in children. The procedure and specific characteristics for Generation R have been described elsewhere (41). For this study we considered mother-reported data on the anxious/depressed, the withdrawn/depressed, and the internalizing problems scales.

### Image Acquisition and Processing

Image acquisition has been described elsewhere (41). In brief, structural brain MR images were obtained on a single 3T GE Discovery MR750w MRI system (General Electric, Milwaukee, WI, USA) utilizing an eight-channel receive-only head coil. T_1_-weighted images were collected using a three-dimensional (3D) inversion recovery-prepared fast spoiled gradient recalled sequence (T_R_ = 8.77 ms, T_E_ = 3.4 ms, T_I_ = 600 ms, flip angle = 10°, field of view = 220 x 220 mm, acquisition matrix = 220 x 220, slice thickness = 1 mm, number of slices = 230, bandwidth = 25 kHz). Diffusion-weighted images consisted of three b_0_ volumes and 35 diffusion directions using an echo planar imaging sequence (T_R_ = 12,500 ms, T_E_ = 72 ms, field of view = 240 x 240 mm, acquisition matrix = 120 x 120, slice thickness = 2 mm, number of slices = 65, b = 900 s/mm^2^).

T_1_-weighted images were processed through the FreeSurfer analysis suite, version 6.0.0 (42). The procedure has been described elsewhere (43). Briefly, non-brain tissue was removed, voxel intensities were normalized for B1 inhomogeneity, whole-brain tissue segmentation was performed, and a surface-based model of the cortex was reconstructed. For each participant we obtained metrics for total brain volume, cortical gray matter volume, cerebrospinal fluid volume, and mean cortical thickness. For analyses of APOE status and the AD PGRS we additionally focused on volumes of the hippocampus, the entorhinal cortex, the middle temporal gyrus, and the parahippocampal gyrus. For the PD PGRS we also considered volumes of the nucleus accumbens, the caudate nucleus, the globus pallidi, and the putamen. Finally, for the FTD PGRS we also looked at the frontal and the temporal lobes, and in particular the volume, the mean thickness, and the surface area. For all lateralized structures we took the mean of both sides.

Diffusion tensor imaging (DTI) images were processed through the FMRIB Software Library (FSL), version 5.0.9 (44). The full procedure is described elsewhere (43). Briefly, non-brain tissue was removed and images were corrected for eddy-current artifacts and translations/rotations resulting from head motion. Diffusion tensors were fitted at each voxel using the RESTORE method from the Camino diffusion MRI toolkit (45). We further performed probabilistic white matter fiber tractography in native space for each participant using the FSL plugin AutoPtx to identify connectivity distributions of a number of well-known fiber bundles (46). Average fractional anisotropy and mean diffusivity values were then computed for each white matter tract. Global measures for fractional anisotropy and mean diffusivity were obtained by performing factor analyses on the tract-specific values (47).

### Lipid Profiles

Lipid profiles of the children were assessed from venous blood acquired during the research visits around the age of 6 years after a 30 min fast. Serum total cholesterol, high-density lipoprotein cholesterol (HDL-c), and triglyceride concentrations were derived with the Roche cobas 8000 analyzer (Roche Diagnostics GmbH, Penzberg, Germany), and low-density lipoprotein cholesterol (LDL-c) was estimated using the Friedewald equation (48). We considered these lipids in relation to APOE status and the AD PGRS as the APOE gene plays a significant role in lipid metabolism (49), whereas we did not have such a prior expectation for PD and FTD.

### Statistical Analysis

Statistical analyses were performed with the R statistical package, version 3.5.2 (R 50). We used multiple linear regression for all outcomes, correcting for age at outcome measurement, sex of the child, maternal education (low, intermediate, or high), and the first 10 genomic components. The latter was done to take into account the underlying genetic structure of the population. The serum lipid models were additionally adjusted for body mass index (BMI) at the time of the venous puncture. The volumetric neuroimaging models, i.e. cortical volume, cerebrospinal fluid (CSF) volume, and the disease-specific regional brain volumes, were additionally adjusted for total brain volume. Furthermore, we applied square-root transformations to the CBCL scales to better satisfy the linearity assumption of linear regression.

Polygenic burden may only affect those whose burden is above a certain threshold, thus leading to non-linearity of an association. We assessed this through two approaches: 1) dichotomization of the top PGRS decile *versus* the rest of the population, 2) fitting restrictive cubic splines on the PGRSs to assess any non-linearity in the association.

Use of PGRS in the Generation R Study requires a critical consideration of ethnicity. The GWAS from which we used the summary statistics were based on populations of European ancestry. Findings from GWAS and by extension PGRS are specific to the ethnicity of the original study population. The Generation R study is based in the city of Rotterdam, where about half of all individuals are of non-European ancestry. We focused our main findings on the complete population, but we additionally stratified our analyses for European ancestry to check for any effects related specifically to ethnicity. We additionally performed sensitivity analyses where we did not correct for the first 10 genomic components, to see whether improper correction for population stratification is relevant for studies on APOE and studies on AD, PD, and FTD PGRS (51).

Multiple testing correction was considered on three levels: 1) the PGRS for AD, PD, or FTD, 2) the PGRS thresholds, and 3) the outcome measures. We did not expect dependence among the PGRS of the neurodegenerative diseases. Therefore, we applied a Bonferroni correction across AD, PD, and FTD. As the PGRS thresholds were strongly intercorrelated as well as some of the outcome measures, we applied a false discovery rate (FDR) correction within a given disease. The p-values reported below are those after the FDR correction.

## Results

### Population Characteristics

**Table 1** shows the characteristics of the total study population and stratified by European ancestry and non-European ancestry. Overall, the most common APOE genotypes were ε3/ε3 (64.9%), ε2/ε3 (11.5%), and ε3/ε4 (19.1%), whereas the other genotypes were much less common, i.e. ε2/ε2 (0.5%), ε2/ε4 (2.3%), and ε4/ε4 (1.6%). These numbers were similar for those with European and non-European ancestry.

**Table 1:** Characteristics of the study population.

Characteristics	All	European ancestry	Non-European ancestry
	(N = 5,490)	(N = 2,651)	(N = 2,839)
APOE genotype (%)			
ε2/ε2	0.5	0.5	0.6
ε2/ε3	11.5	10.4	12.6
ε2/ε4	2.3	2.5	2.1
ε3/ε3	64.9	60.6	69.0
ε3/ε4	19.1	21.6	16.8
ε4/ε4	1.6	2.2	1.1
Visit around 6 years			
Non-verbal IQ (mean, SD)	101 (15)	105 (14)	97 (15)
Total cholesterol (mean, SD) (mmol/L)	4.2 (0.6)	4.2 (0.6)	4.3 (0.7)
HDL-c (mean, SD) (mmol/L)	1.3 (0.3)	1.3 (0.3)	1.4 (0.3)
LDL-c (mean, SD) (mmol/L)	2.4 (0.6)	2.3 (0.6)	2.4 (0.6)
Triglycerides^a^ (geometric mean, SD) (mmol/L)	1.0 (0.5)	1.1 (0.5)	1.5 (0.5)
Visits at 10 and 11 years			
CITO score, standardized (mean, SD)	0.0 (1.0)	0.2 (0.9)	−0.3 (1.1)
Total brain volume (mean, SD) (cm^3^)	1,200 (118)	1,225 (113)	1,168 (116)
Cortical volume (mean, SD) (cm^3^)	574 (59)	588 (56)	556 (59)
Cerebrospinal fluid volume (mean, SD) (cm^3^)	0.9 (0.2)	0.9 (0.2)	0.9 (0.2)
Mean cortical thickness (mean, SD) (mm)	2.67 (0.08)	2.68 (0.08)	2.67 (0.08)
Global FA, standardized (mean, SD)	0.00 (1.00)	0.11 (0.97)	−0.15 (1.02)
Global MD, standardized (mean, SD)	0.00 (1.00)	−0.03 (0.98)	0.04 (1.03)
Anxious/depressed scale (mean, SD)	2.2 (2.7)	2.2 (2.7)	2.2 (2.6)
Withdrawn/depressed scale (mean, SD)	1.1 (1.6)	1.1 (1.5)	1.1 (1.8)
Internalizing problems scale (mean, SD)	4.7 (5.0)	4.5 (4.8)	5.1 (5.4)

APOE, apolipoprotein epsilon.

IQ, intelligence quotient.

HDL-c, high density lipoprotein cholesterol.

LDL-c, low density lipoprotein cholesterol.

FA, fractional anisotropy.

MD, mean diffusivity.

a Triglyceride serum values were log-transformed.

### Apolipoprotein E and Polygenic Risk Scores for Alzheimer’s Disease

**Figure 1:**
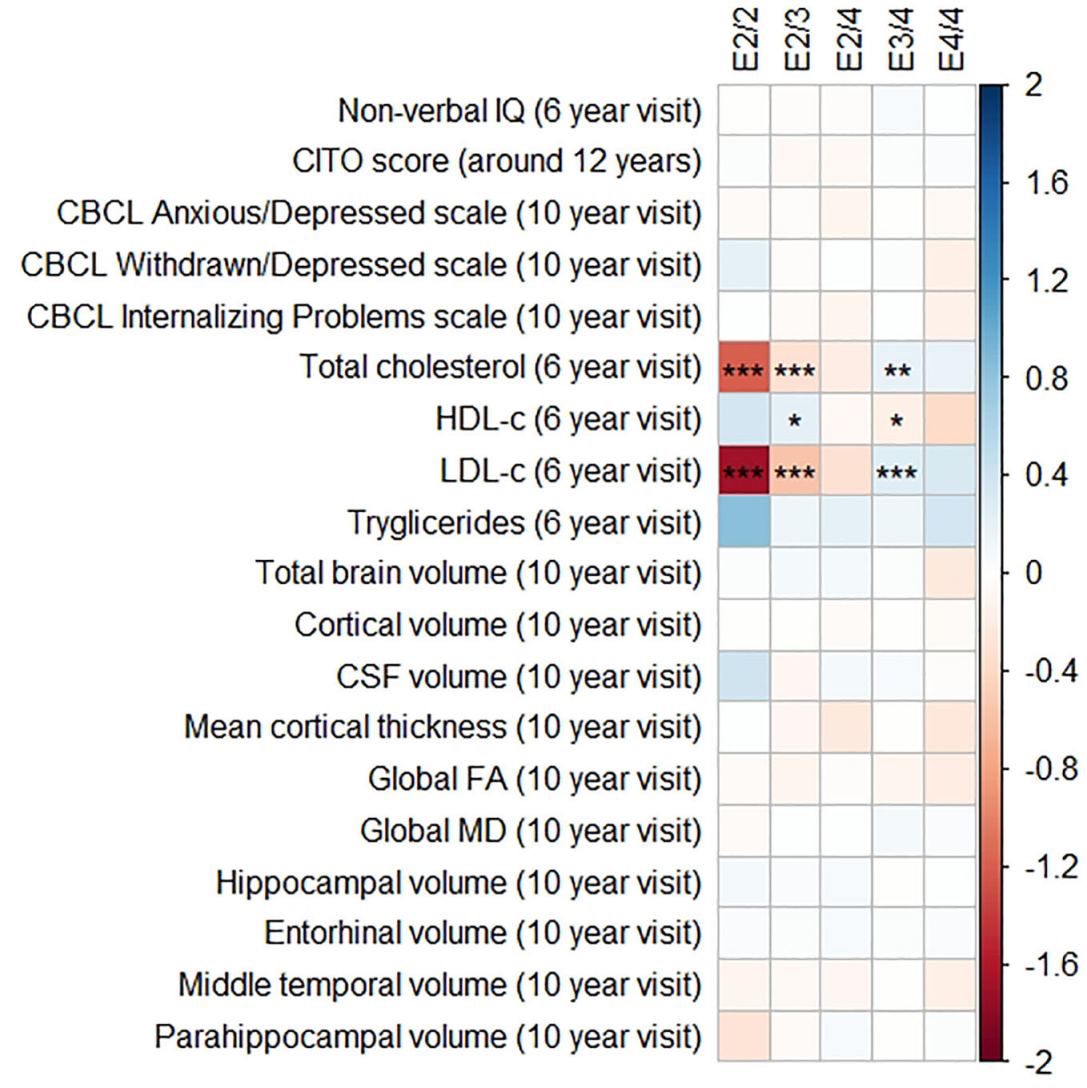
Heatmap showing the regression coefficients between apolipoprotein E (APOE) genotype and all phenotypes. The E3/3 genotype is used as a reference for the other genotypes. All coefficients are standardized. The reported p-values were corrected for multiple testing. IQ, intelligence quotient; CBCL, Child Behavior Checklist; HDL, high-density lipoprotein; LDL, low-density lipoprotein; CSF, cerebrospinal fluid; FA, fractional anisotropy; MD, mean diffusivity; * = 0.05; ** = 0.01; *** = 0.001.

**Figure 2:**
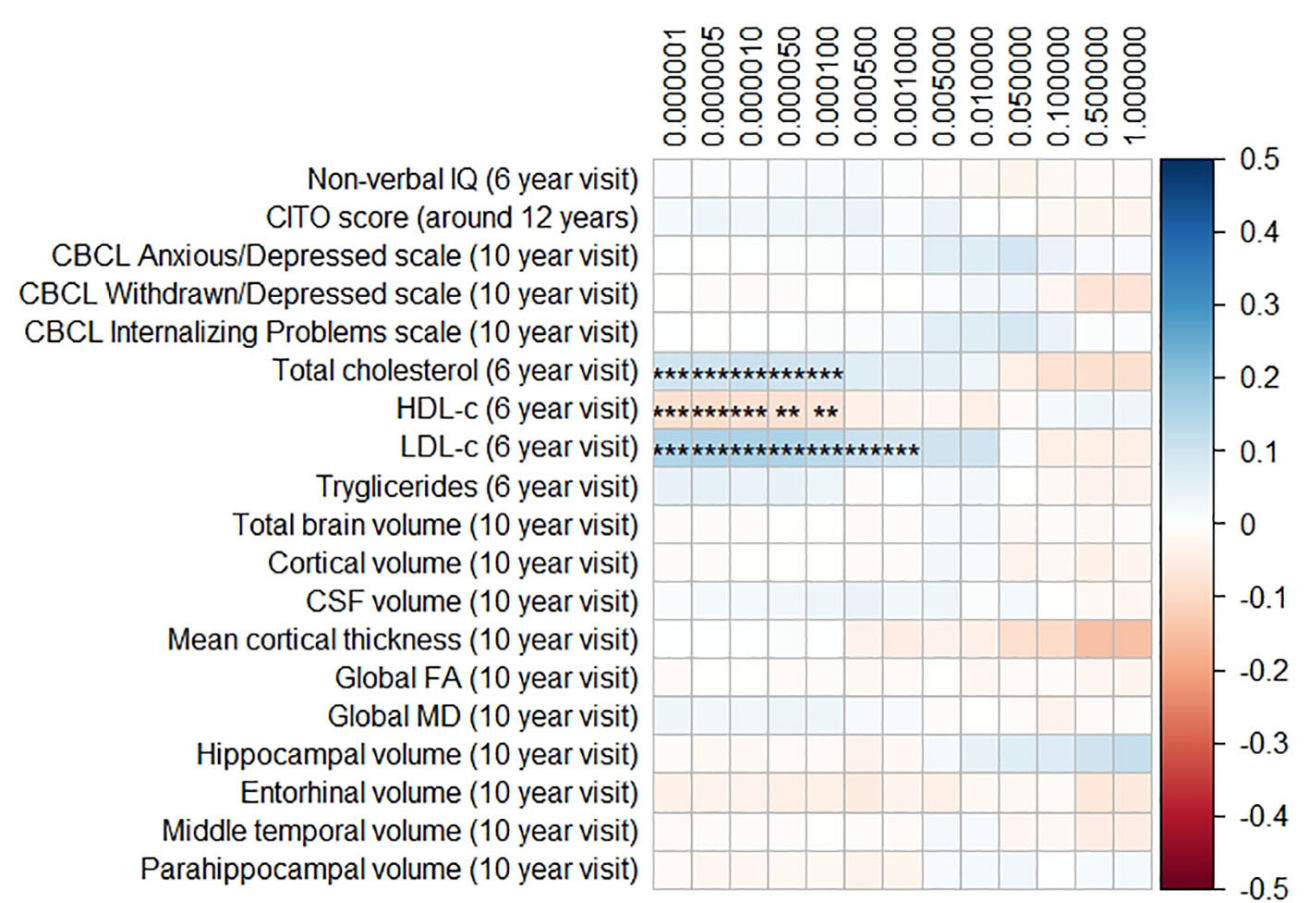
Heatmap showing the regression coefficients between the Alzheimer’s disease (AD) polygenic risk score (PGRS) and all phenotypes. Each score is based on a different threshold for inclusion of single-nucleotide polymorphisms (SNPs) into the score. All coefficients are standardized. The reported p-values were corrected for multiple testing. IQ, intelligence quotient; CBCL, Child Behavior Checklist; HDL, high-density lipoprotein; LDL, low-density lipoprotein; CSF, cerebrospinal fluid; FA, fractional anisotropy; MD, mean diffusivity; ** = 0.01; ** = 0.001.

**Figures 1** and **2** display the results of the associations of all relevant outcomes with APOE genotype and AD PGRS, respectively. Neither APOE genotype nor any AD PGRS associated with non-verbal IQ during the 6-year visit or the CITO score at 11 years (all p_corrected_ > 0.05). The APOE genotype and AD PGRS also did not relate to global brain metrics such as total brain volume and CSF volume, nor with the connectivity metrics global fractional anisotropy and mean diffusivity (all p_corrected_ > 0.05). APOE genotype and the AD PGRS also did not associate with region-specific metrics for the hippocampus, the entorhinal cortex, the medial temporal gyrus, and the parahippocampal region. Finally, APOE genotype and AD PGRS did not show any statistically significant associations with the CBCL scales anxious/depressed or withdrawn/depressed, nor with the internalizing problems scale (all p_corrected_ > 0.05).

The APOE genotype associated with serum lipid profiles. Compared to the APOE ε3/ε3 genotype, those with APOE ε2/ε3 had lower total cholesterol concentrations (β = −0.32, SE = 0.06, p_corrected_ < 0.001), lower LDL-c concentrations (β = −0.57, SE = 0.06, p_corrected_ < 0.001), and higher HDL-c concentrations (β = 0.22, SE = 0.06, p_corrected__=_ 0.01). The APOE ε2/ε2 followed the exact same pattern but with even larger differences.

Compared to the APOE ε3/ε3 genotype those with ε3/ε4 had higher total cholesterol concentrations (β = 0.19, SE = 0.05, p_corrected_ = 0.003), higher LDL-c concentrations (β = 0.16, SE = 0.05, p_corrected_ < 0.001), and lower HDL-c concentrations (β = 0.26, SE = 0.05, p_corrected_ = 0.02). These differences were similar and larger when comparing the APOE ε3/ε3 genotype with the APOE ε4/ε4 genotype.

Triglycerides were higher in all genotypes compared to APOE ε3/ε3, although none of these were statistically significance (all p_corrected_ > 0.05).

The AD PGRS also associated with serum lipid profiles, but only at stricter PGRS thresholds, i.e. PGRS thresholds below 0.001. However, these associations disappeared upon including the APOE genotype as a covariate (all p_corrected_ > 0.05). Furthermore, the results did not differ when using the top-decile PGRS dichotomization rather than the continuous PGRS, or when modeling cubic splines.

### Polygenic Risk Scores for Parkinson’s Disease and Frontotemporal Dementia

**Figure 3:**
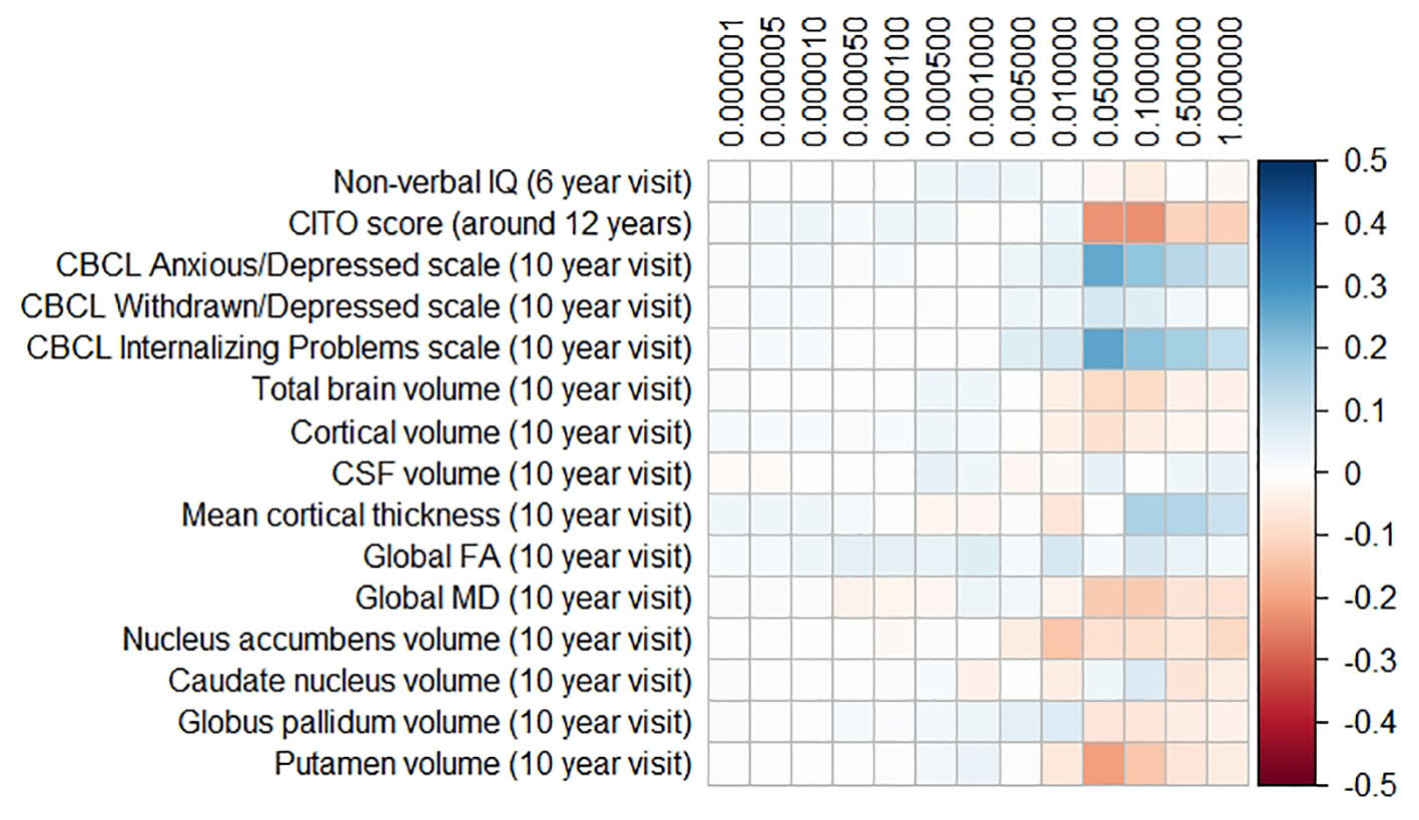
Heatmap showing the regression coefficients between the Parkinson’s disease (PD) polygenic risk score (PGRS) and all phenotypes. Each score is based on a different threshold for inclusion of single-nucleotide polymorphisms (SNPs) into the score. All coefficients are standardized. The reported p-values were corrected for multiple testing. None of the associations were statistically significant after correction. IQ, intelligence quotient; CBCL, Child Behavior Checklist; HDL, high-density lipoprotein; LDL, low-density lipoprotein; CSF, cerebrospinal fluid; FA, fractional anisotropy; MD, mean diffusivity.

**Figure 4:**
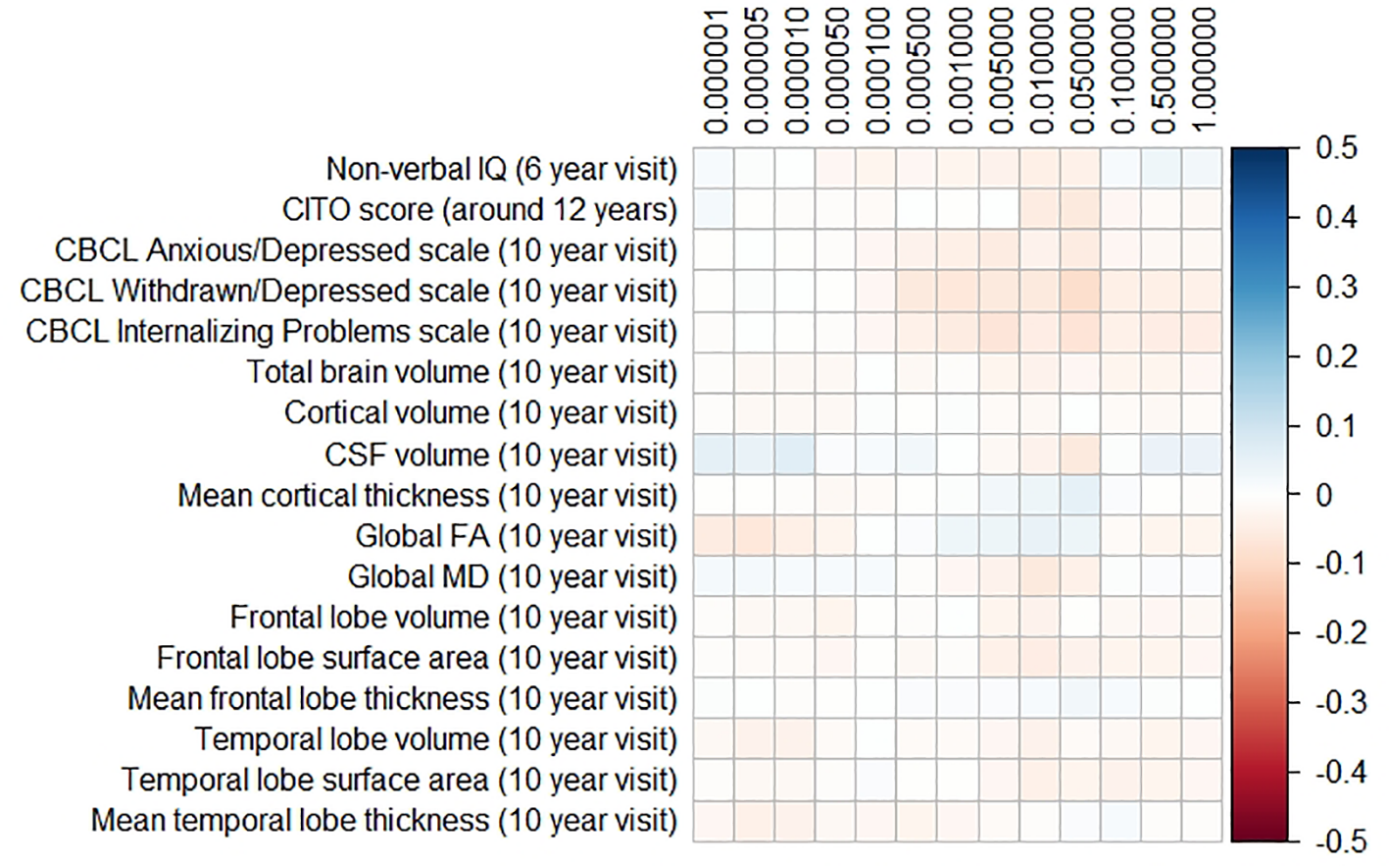
Heatmap showing the regression coefficients between the frontotemporal dementia (FTD) polygenic risk score (PGRS) and all phenotypes. Each score is based on a different threshold for inclusion of single-nucleotide polymorphisms (SNPs) into the score. All coefficients are standardized. The reported p-values were corrected for multiple testing. None of the associations were statistically significant after correction. IQ, intelligence quotient; CBCL, Child Behavior Checklist; HDL, high-density lipoprotein; LDL, low-density lipoprotein; CSF, cerebrospinal fluid; FA, fractional anisotropy; MD, mean diffusivity.

The results for the PD and FTD PGRS are shown in **Figures 3** and **4**, respectively. We found no support for associations of scores at any threshold with non-verbal IQ, educational attainment, internalizing behavior scales, or neuroimaging markers. Furthermore, we did not find evidence for associations of the PD scores with the volumes of the nucleus accumbens (ß for PGRS at 0.05 threshold = −0.08, SE_0.05_ = 0.12, p_corrected_ = 1.00), the caudate nucleus (ß_0.05_ = 0.03, SE_0.05_ = 0.12, p_corrected__=_ 1.00), the globus pallidus (ß_0.05_ = −0.07, SE_0.05_ = 0.13, p_corrected_ = 1.00), or the putamen (ß_0.05_ = −0.13, SE_0.05_ = 0.12, p_corrected_ = 1.00). Similarly, we did not observe any associations of the FTD scores with the volumes of the frontal (ß_0.05_ = −0.00, SE_0.05_ = 0.03, p_corrected_ = 1.00) or temporal lobes (ß_0.05_ = 0.01, SE_0.05_ = 0.03, p_corrected_ = 1.00).

### Population Structure

**Figure 5:**
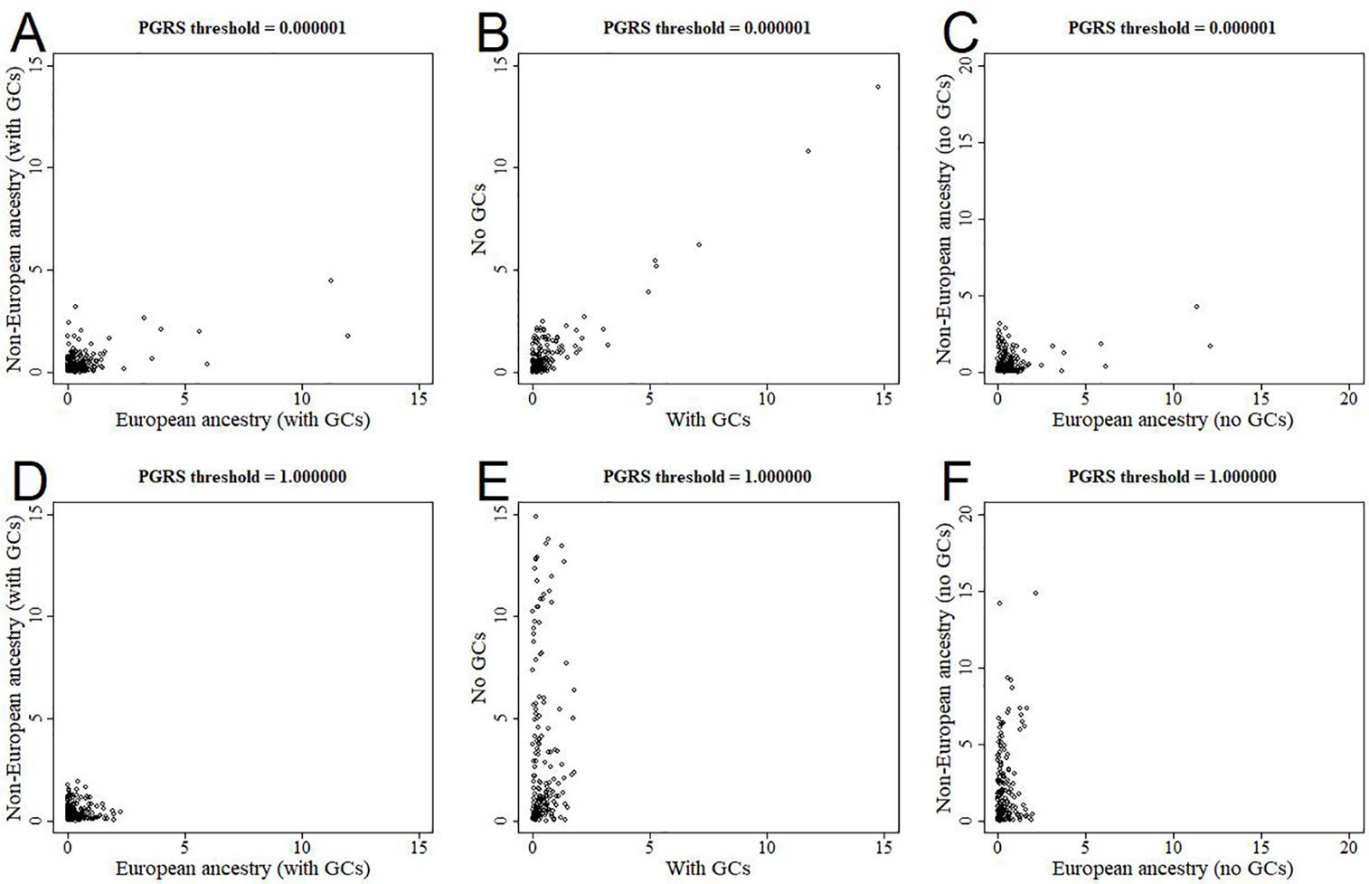
Scatterplots of log-transformed p-values when comparing different analyses. The results are shown for polygenic risk score (PGRS) thresholds 0.000001 (top row) and 1.000000 (bottom row). Comparisons are shown for European *versus* non-European **(A, D)**, correcting for genomic components or not **(B, E)**, and European *versus* non-European when not correcting for genomic components **(C, F)**. GC, genomic component.

All analyses were performed in all available participants, and were controlled for the first 10 genomic components. We further stratified the analyses for European *versus* non-European ancestry (**Figures 5A, D**, **Supplementary Figure 2**), and the effect estimates were generally similar. We additionally reran the analyses without correcting for the genomic components, and this led to stark changes in the results (**Figures 5B, E**, **Supplementary Figure 3**). The higher the PGRS threshold, the more statistically significant findings were present in the analyses not corrected for genomic components compared to when we did correct for genomic components. We further split the uncorrected analyses for European *versus* non-European ancestry, to see whether one of these groups was driving the sudden change in findings (**Figures 5C, F**, **Supplementary Figure 4**). Within the uncorrected analyses for individuals of non-European ancestry we find an inflation of the number of statistically significant findings, whereas this was not the case for individuals of European ancestry.

## Discussion

None of the measures for genetic burden for AD, PD, or FTD were associated with childhood non-verbal IQ, educational attainment, internalizing behavior, global brain structure, or disease-specific regional brain structures. Although genetic burden for late-life neurodegenerative disease has been linked to brain structure and cognitive function during late-life, we find no evidence that these affect the same processes during early life. Furthermore, we provided clear evidence that the APOE genotype affects lipid profiles during childhood. Finally, we showed that improper control of the ethnic structure of the population through genomic components can lead to false positive associations when considering PGRS for AD, PD, and FTD.

We found no support for the link between AD genetic burden and global, hippocampal. and temporal regions although previous studies have provided evidence for such links during infancy (28, 29), childhood (25, 30, 52, 53), and early adulthood (27, 54–61). The support for such associations does seem stronger in studies during early adulthood than during childhood. This suggests that the genetic burden for AD becomes more relevant with age, and that there are cumulative processes at play which may only become apparent after early-life. For example, the pathological burden in APOE ε4 carriers may be increased due to accumulation of lipoprotein (62, 63), reduced neuronal reparative capacity (64, 65), and altered responses to neuroinflammatory processes (66).

Interpreting the role of APOE in childhood brain development is further complicated by the inconsistency of findings. Shaw and colleagues found in children and adolescents that APOE ε4 carriers had thinner entorhinal cortices than non-carriers (25). Chang and colleagues also studied children and adolescents, but they found that ε4 carriers had larger hippocampi than non-carriers (30). Additionally, they report that ε4 carriers compared to non-carriers had larger volumes for the cuneus, the temporal pole, the lateral occipital pole, and the medial orbitofrontal cortex. The two studies that report that APOE affects brain structure in infants show that ε4 carriers have smaller hippocampi than non-carriers (28, 29). Furthermore, they report that APOE affects regions that are very different from those reported by Chang and colleagues (30). More recently, Axelrud and colleagues reported that the AD PGRS relates to hippocampal volume in Brazilian children aged 6 to 14 years old (53). However, the source GWAS on which the PGRS in the latter study was based was performed in a population of European ancestry (37). As we have shown, using the AD PGRS in populations of non-European ancestry leads to false positive findings. Indeed, Axelrud and colleagues could not replicate their findings in a separate Canadian population of 1,024 adolescents. In summary, previous findings have been inconsistent, which suggests that AD genetic burden may only affect early-life brain structure under specific circumstances or that the effect is unlikely to be clinically relevant.

We did not find evidence in our study to suggest that AD genetic burden affects cognitive functioning during childhood. Previous studies on this topic report mixed results, but several larger studies also did not find evidence for such a link. Taylor and colleagues studied cognition in the Avon Longitudinal Study of Parents and Children (ALSPAC) study (23). The only pattern observed was that APOE ε4 carriers performed better on cognitive tests than those with a APOE ε3/ε3 genotype, although not statistically significant. In our study we found no evidence to support this. More recently, Weissberger and colleagues meta-analyzed data from 9,234 individuals aged 2 to 40 years old and found no association of APOE ε4 carriership with intelligence, attention, executive function, language, memory, processing speed, and visuospatial abilities (67). In our study we confirmed this finding at two timepoints in childhood (around 6 years of age and around 11 years of age), and we also extend the findings to APOE ε2 genotypes and to broader AD genetic burden. Taken together, the literature and this study suggest that AD genetic burden does not affect cognition during early life.

The role of the APOE gene in serum lipid profiles during early life has been studied before. In 2011, a study by Taylor and colleagues assessed the relation between APOE status and serum lipid profiles in 2,875 children aged 8 to 11 years from the ALSPAC cohort, a prospective birth cohort study (23). They showed that carriership of APOE ε2 was associated with reduced cholesterol and increased triglyceride levels compared to APOE ε3/ε3, whereas APOE ε4 carriers had both elevated cholesterol and triglyceride levels. In 1997, Kallio and colleagues showed that cord blood from 42 APOE ε4 carriers contained higher concentrations of cholesterol than 13 carriers of APOE ε2 (22). In addition, LDL levels rose steeper during the first year of life in the APOE ε4 carriers than in the APOE ε2 carriers. In our study we had similar findings for ε2/ε3 and ε3/ε4 but not for ε2/ε2 and ε4/ε4 genotypes, likely because those genotypes were uncommon within the current study population. Our findings further consolidate the causal role of APOE genotype in serum lipid levels even during early life.

We found no evidence that PD and FTD genetic burden influences early-life processes. However, the etiology and pathogenesis of PD and FTD are poorly understood, and less is known on the preclinical disease stage compared to AD. It is therefore not clear how genetic burden for PD or FTD would influence early-life processes. As both syndromes can occur through dominant autosomal inheritance, it should be possible to investigate families of PD or FTD patients to identify such processes. However, we were unable to identify any such study in the literature. Another route would be to look at healthy carriers of known genetic risk variants for either PD or FTD to identify affected processes. For example, the G2019S mutation in the LRRK2 gene, the gene most widely associated with Parkinson’s disease, has been studied in healthy controls. Different studies found this gene to be associated with lower executive functioning (68), changes in gait (69), olfactory dysfunction (70). However, all these studies were small and exploratory. To the best of our knowledge, no studies focusing on FTD candidate genes in healthy controls are available. Further work is needed to elucidate whether PD and FTD genetic burden play a role in other domains during early-life, for example brain function rather than brain structure.

The etiology of AD, PD, and FTD extend beyond lipid profiles, the brain, behavior, and cognition, thus raising the question which other processes could be relevant during childhood. For example, cerebrospinal fluid markers levels such as Tau and phosphorylated Tau are affected by APOE ε4 carriership in demented individuals (71–74). In addition, APOE protein levels in cerebrospinal fluid, but not blood serum, depend on the APOE genotype (75). Another avenue for further research are inflammatory markers such as C-reactive protein, interleukin-6, and α1-antichymotrypsin, which have shown predictive value for the onset of all-cause dementia (76). Further assessment of endophenotypes closely related to specific gene function may provide more stable findings related to early life.

Our findings may have been limited by several aspects of the study design. First, we relied on cross-sectional data. Brain growth follows non-linear trajectories, reaching a peak at around the age when the children in our study underwent neuroimaging (77). The genetic burdens for neurodegenerative disease may affect the trajectories of brain development, which would only be detectable through longitudinal studies. Alternatively, the genetic burden for late-life neurodegenerative disease may not express until later in childhood or adolescence, and the study population may simply be too young for the research questions at hand. Second, the number of individuals with ε2/ε2 or ε4/ε4 genotypes was relatively low, thus we were likely underpowered to establish any small effects for those genotypes. Third, we administered a limited number of cognitive tests around the age of 6, limiting our investigation to non-verbal IQ. AD is generally characterized by a loss of memory function, for which we did not have an adequate test in children.

Our study also had clear strengths. The size of our study population ensured sufficient power to detect relatively small effects related to the common APOE genotypes and the AD, PD, and FTD PGRS. Furthermore, we provide an unambiguous case for proper control of population stratification, which was only possible due to the large proportion of participants of non-European ancestry. Finally, the Generation R study is a representative sample from the general population, which vastly improves the generalizability to a community-dwelling population of European descent.

In conclusion, we found no evidence to support the role of genetic burden for late-life neurodegenerative disease in early-life cognitive performance, internalizing behavior, and brain metrics. APOE genotype was related to blood lipid profiles. Genetic burden for AD, PD, and FTD did not relate to cognition or brain structure. These findings suggest that the etiology of late-life neurodegenerative disease becomes only relevant later in life.

## Data Availability Statement

The datasets for this article are not automatically publicly available due to legal and informed consent restrictions. Reasonable requests to access the datasets should be directed to the Director of the Generation R Study, Vincent Jaddoe (generationr@erasmusmc.nl), in accordance with the local, national and European Union regulations.

## Author Contributions

SL, RM, TW, and HA designed the project. SL analyzed the data. SL and HA interpreted the results and drafted the article. All authors contributed to manuscript revision, and read and approved the submitted version. All authors agree to be accountable for all of the published work.

## Funding

The work was supported by the European Research Council (ERC) under the European Union’s Horizon 2020 research and innovation program (project: ORACLE, grant agreement No: 678543, and project JPco-fuND, grant agreement No: 643417), the ZonMw (grant numbers 912.11.021 and 916.19.151), and the Sophia Foundation (grant S18-20). The general design of Generation R Study is made possible by financial support from the Erasmus Medical Center, Rotterdam, the Erasmus University Rotterdam, the Netherlands Organization for Health Research and Development (ZonMw), the Netherlands Organisation for Scientific Research (NWO), the Ministry of Health, Welfare and Sport, and the Ministry of Youth and Families.

## Conflict of Interest

The authors declare that the research was conducted in the absence of any commercial or financial relationships that could be construed as a potential conflict of interest.

## Supplementary Material

Supplementary Figure 1 Flow chart of the study population.

Supplementary Figure 2 Scatterplots of log-transformed p-values when comparing analyses in the European versus non-European samples when correcting for genomic components.

Supplementary Figure 3 Scatterplots of log-transformed p-values when comparing analyses corrected for genomic components versus not.

Supplementary Figure 4 Scatterplots of log-transformed p-values when comparing analyses in the European versus non-European samples when not correcting for genomic components.
