# Supplementary figures and images for: Genetic Burden for Late-Life Neurodegenerative Disease and Its Association With Early-Life Lipids, Brain, Behavior, and Cognition
Source: Front Psychiatry. 2020 Feb 7;11:33. doi: 10.3389/fpsyt.2020.00033 (PMC7018686; doi:10.3389/fpsyt.2020.00033)

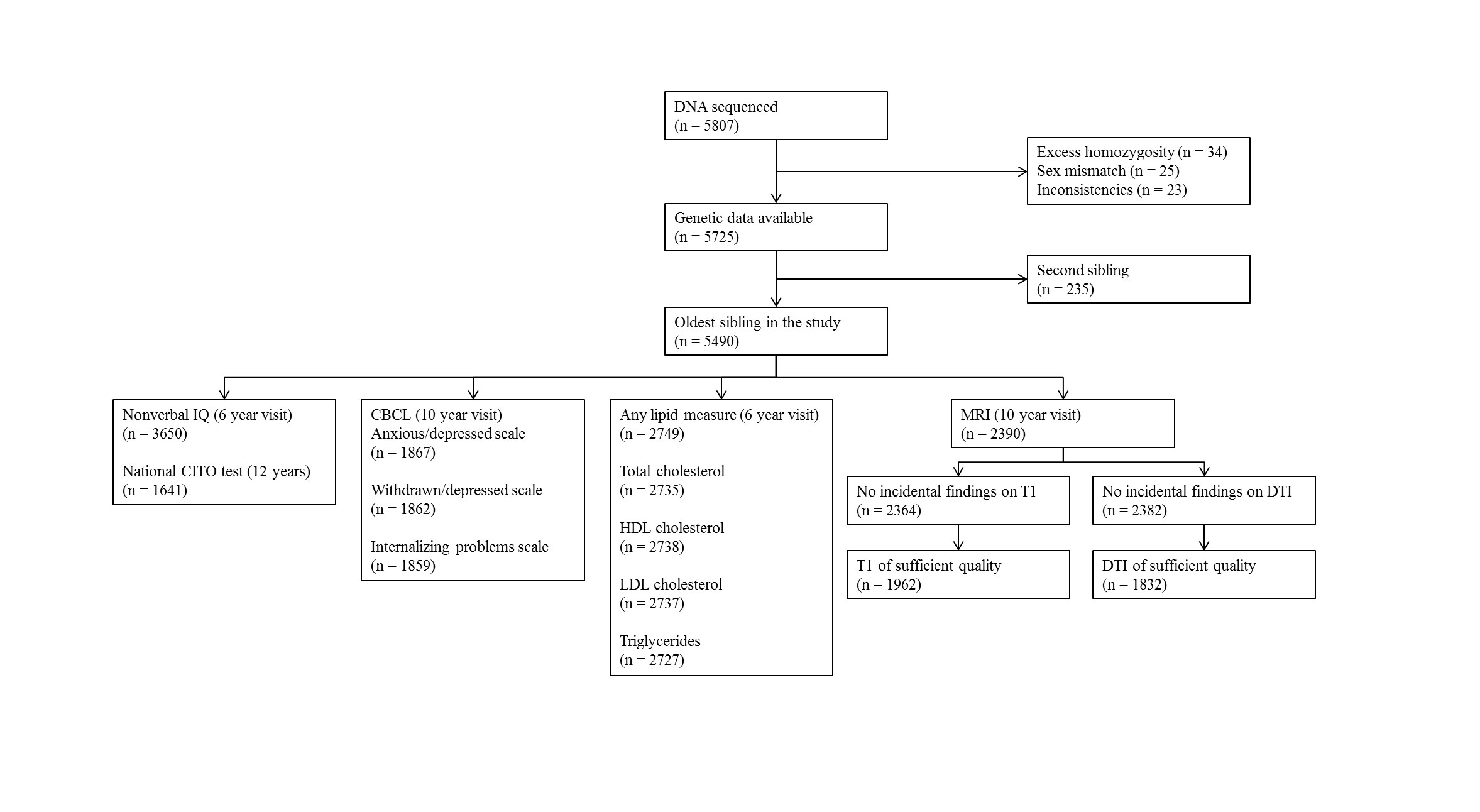

Supplement: Supplementary Figure 1 — Flow chart of the study population. [file Image_1.jpeg]

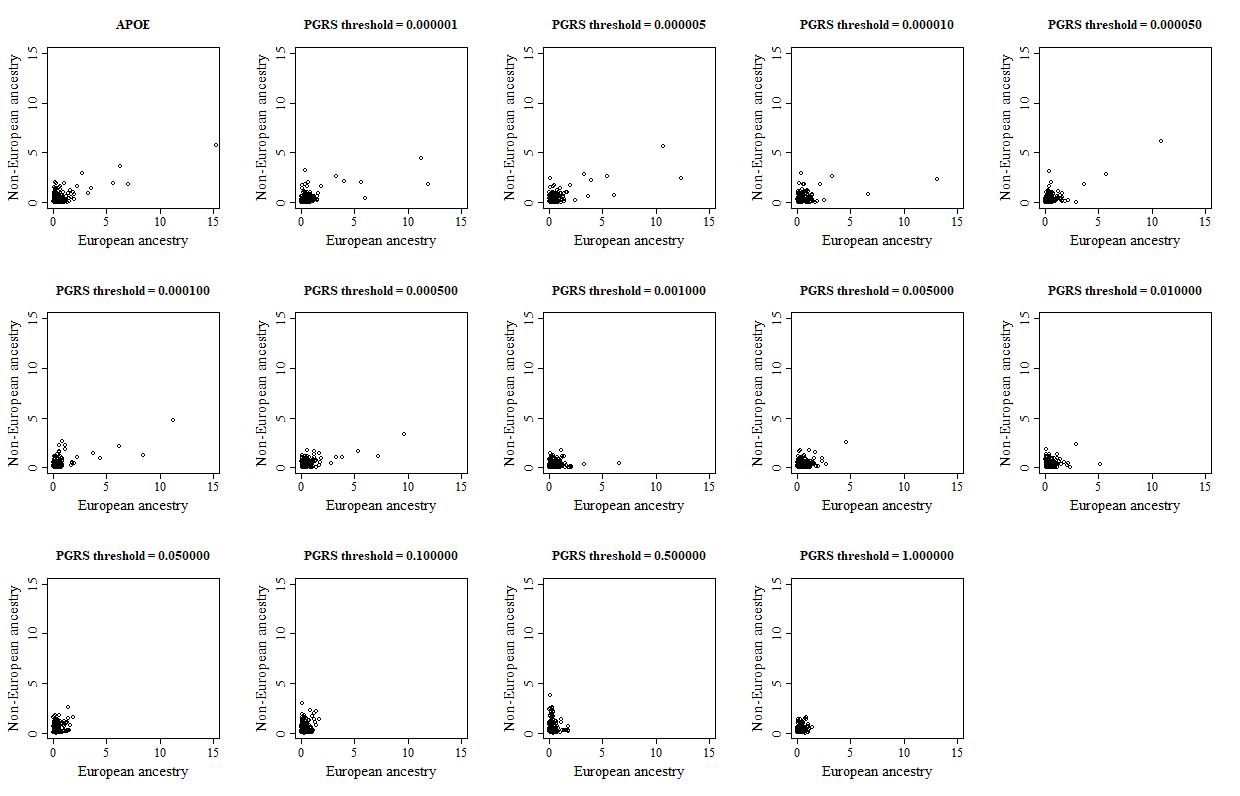

Supplement: Supplementary Figure 2 — Scatterplots of log-transformed p-values when comparing analyses in the European versus non-European samples when correcting for genomic components. [file Image_2.jpeg]

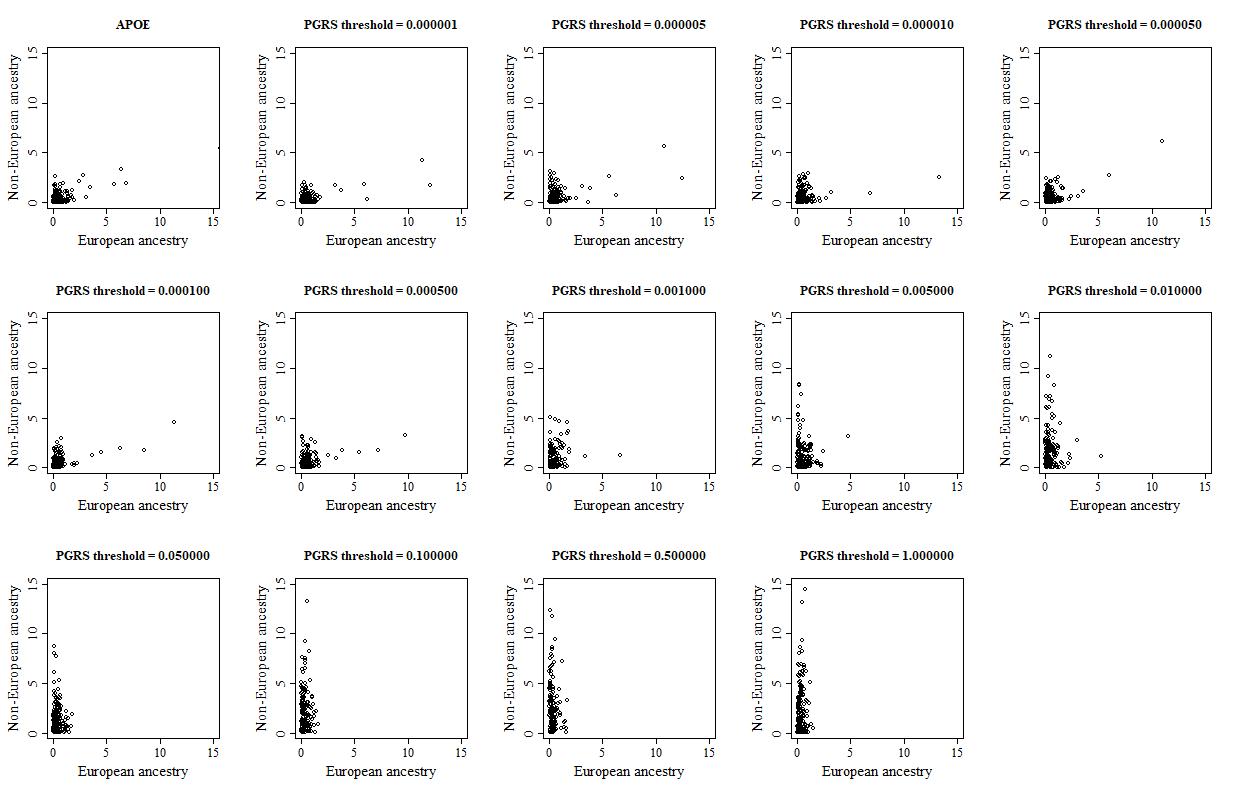

Supplement: Supplementary Figure 3 — Scatterplots of log-transformed p-values when comparing analyses corrected for genomic components versus not. [file Image_3.jpeg]

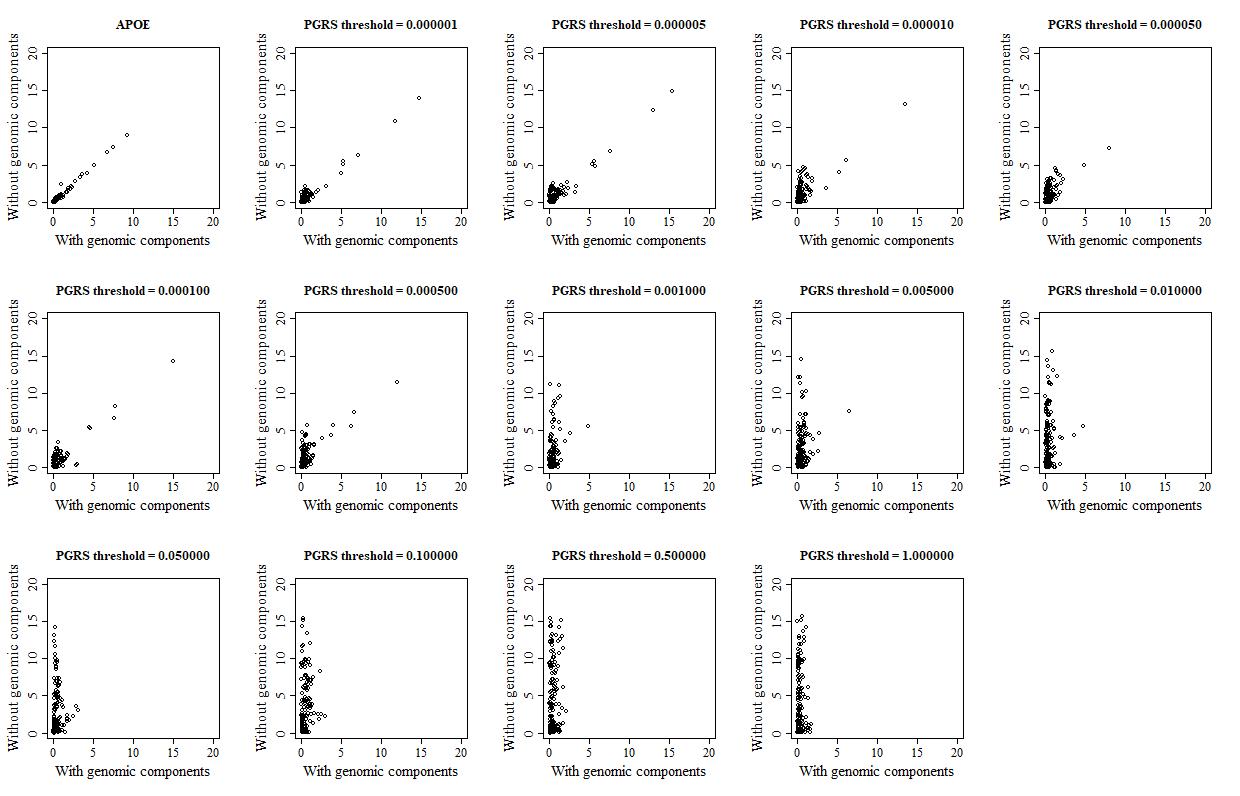

Supplement: Supplementary Figure 4 — Scatterplots of log-transformed p-values when comparing analyses in the European versus non-European samples when not correcting for genomic components. [file Image_4.jpeg]
